# Extracellular matrix molecules and their potential contribution to the function of transplanted pancreatic islets

**DOI:** 10.1007/s00125-017-4524-8

**Published:** 2018-01-06

**Authors:** L. Alberto Llacua, Marijke M. Faas, Paul de Vos

**Affiliations:** 1 Section of Immunoendocrinology, Department of Pathology and Medical Biology, University of Groningen, Hanzeplein 1 EA11, 9700 RB Groningen, the Netherlands; 2 University Medical Center Groningen, University of Groningen, Groningen, the Netherlands

**Keywords:** Collagen, Cytokines, Extracellular matrix, Graft, Islet, Laminin, Review

## Abstract

Extracellular matrix (ECM) molecules are responsible for structural and biochemical support, as well as for regulation of molecular signalling and tissue repair in many organ structures, including the pancreas. In pancreatic islets, collagen type IV and VI, and laminins are the most abundant molecules, but other ECM molecules are also present. The ECM interacts with specific combinations of integrin α/β heterodimers on islet cells and guides many cellular processes. More specifically, some ECM molecules are involved in beta cell survival, function and insulin production, while others can fine tune the susceptibility of islet cells to cytokines. Further, some ECM induce release of growth factors to facilitate tissue repair. During enzymatic isolation of islets for transplantation, the ECM is damaged, impacting islet function. However, restoration of the ECM in human islets (for example by adding ECM to the interior of immunoprotective capsules) has been shown to enhance islet function. Here, we provide current insight into the role of ECM molecules in islet function and discuss the clinical potential of ECM manipulation to enhance pancreatic islet function and survival.

**Electronic supplementary material:**

The online version of this article (10.1007/s00125-017-4524-8) contains a slideset of the figures for download, which is available to authorised users.

## Introduction

Type 1 diabetes is an autoimmune disorder leading to the destruction of insulin-producing beta cells. Current clinically available methods of insulin replacement cannot prevent the occurrence of frequent hypoglycaemia and diabetic complications [1]. Hence, an insulin source that regulates glucose levels on a minute-by-minute basis to prevent hypoglycaemia and diabetic complications [2, 3] and to improve quality of life and life expectancy [1, 4, 5] is required. Theoretically, this can be achieved by transplantation of allogeneic pancreatic islets (either via transplantation of the whole pancreas or isolated pancreatic islets) [4, 6]. Both whole pancreas or pancreatic islet transplantation prevents the development of hypoglycaemia and diabetic complications [7], but the latter has two principle advantages over the former. First, islets can be modulated before transplantation to reduce the risk of graft rejection. Second, islet transplantation is a minimally invasive surgical procedure, involves a short hospital stay, has low morbidity [4] and can be repeated with minor adverse effects in case of graft failure.
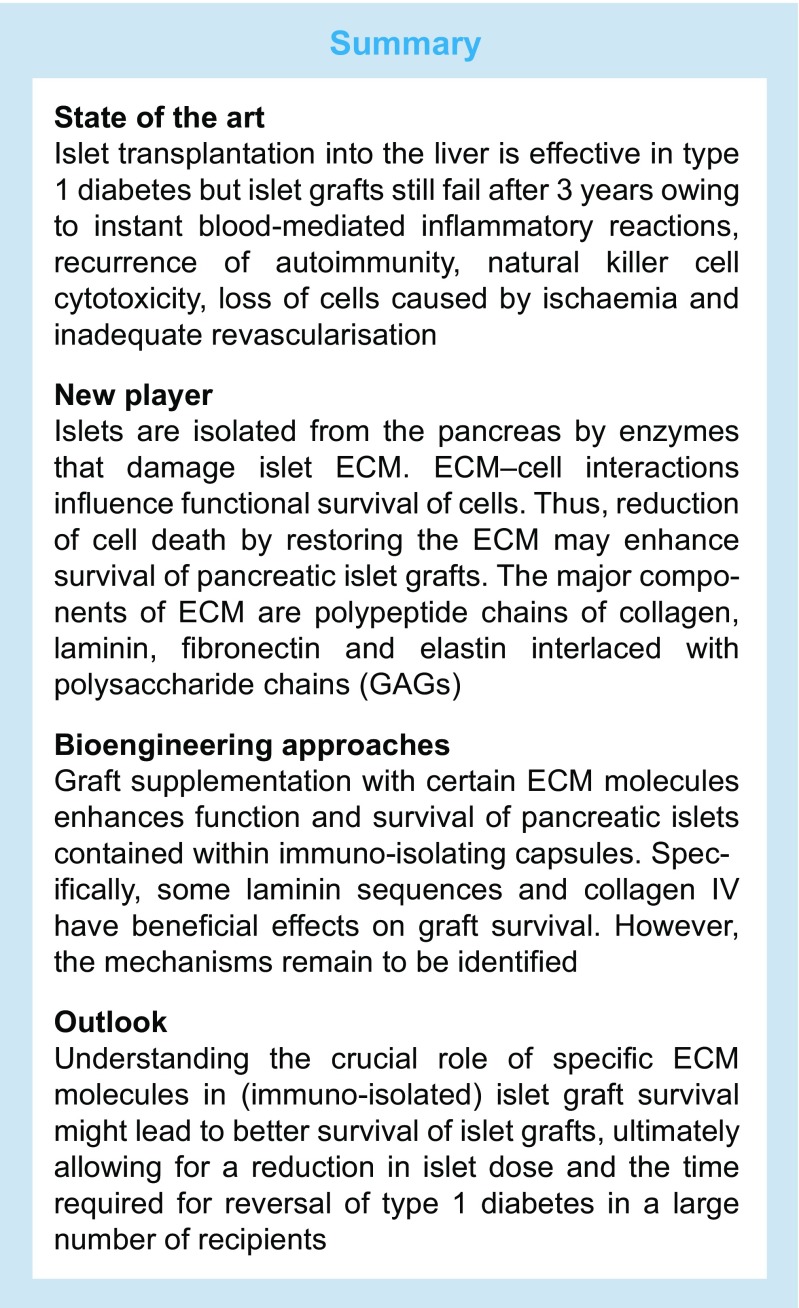


Islets are conventionally transplanted by infusion into the liver via the portal vein. This procedure is clinically effective in type 1 diabetes [8] but islet grafts often fail after 3–5 years despite the use of immunosuppressants. Many factors contribute towards graft failure, including instant blood-mediated inflammatory reactions (IBMIRs) [9], recurrence of autoimmunity [10], natural killer (NK) cell cytotoxicity [2, 11, 12], loss of cells owing to ischaemia [13] and inadequate revascularisation [13, 14].

Recently, the importance of the extracellular matrix (ECM) in islet transplantation has been recognised [3, 6, 15, 16], although the precise role of islet ECM integrity in graft function and survival is not yet understood. Pancreatic islets have an extensive network of ECM molecules [17–20]; these are damaged during isolation of pancreatic islets via application of enzymes that break down ECM molecules between endocrine and exocrine cells [6, 21]. Although selective, the process of enzymatic degradation of exocrine connections is not specific [2] and, as a consequence, many ECM components that surround the islets and interconnect endocrine cells are also damaged [2, 22], affecting islet function [6, 21, 23, 24]. After isolation using ECM-degrading collagenases, the whole microvasculature of the islet is destroyed [25] and islet cells undergo cell-death processes, such as anoikis, necroptosis and necrosis [2, 22, 26, 27]. To amplify matters, these processes are associated with the release of highly inflammatory danger-associated molecular patterns (DAMPs) that contribute to immune responses against pancreatic islets [28]. Therefore, it is conceivable that islet cell death may be reduced via restoration of the ECM to enhance survival of pancreatic islet grafts. In fact, graft supplementation with ECM molecules has been shown to enhance the function and survival of pancreatic islets (via mechanisms that are largely unknown) [3, 18, 29]. The applicability of this approach has already been demonstrated under settings of tissue engineering, whereby ECM supplementation has contributed to the success of grafts [23, 30], with the supplemented ECM guiding cellular development by mimicking the biochemical composition, fibrillar structure and viscoelastic properties of the ECM in the target organ [31].

Here, we provide a summary of the types of ECM molecules that are normally expressed in the islet, followed by a discussion of the current knowledge on the contribution made by ECM to the function of islets and other organs. This knowledge will facilitate the future design of islet ECM complexes applicable for islet transplantation. We conclude by discussing the current experimental proof of principle of improving islet function by supplementation with specific ECM components in immuno-isolating islet grafts.

## ECM structures that might support islet function

ECM molecules play an important role in guiding proliferation, differentiation and migration of cells, starting as early as embryogenesis [32, 33]. They also modulate and attenuate inflammatory responses in various organs [34]. Usually, collagens provide structural stiffness and cohesiveness to tissues [24, 32, 35] and laminin chains may be critical in maintaining the integrity and shape of an organ structure [32, 36]. Moreover, fibronectin, fibrillin and laminin are found in the pancreas and are involved in cytoskeletal remodelling, contractility and differential cell adhesion [32, 37]. Pancreatic islets contain almost all major ECM molecules in varying proportions [18, 19, 22, 38]. The most abundant ECM molecules in pancreatic islets are collagen type IV and VI, and laminins, such as laminin-332 and laminin-511 [39–41]. Most ECM molecules in the islets have been associated with specific biological processes, as discussed below. However, some ECM molecules in the pancreatic islets have not been studied to a great extent and, thus, are yet to be fully characterised; for these, we will describe their function in other organs, where they likely perform similar functions as in the islets.

### Collagen

Collagens are the most abundant protein in organisms and are usually divided into fibrillar and nonfibrillar structures [42, 43]. Over 20 chemically distinct collagen structures have been described. Collagens I, II, III, IV, V and VI are present in the peripheral ECM of mature human islets but only collagens type I and IV are commonly used as supplements/adjuvants for cellular functions in biomedical applications [44, 45].

Collagens VI and IV are located at the islet–exocrine interface and islet basement membrane and regulate fibronectin assembly by restraining cell–fibronectin interactions [46–48]. Collagen IV is also abundant in the peripheral matrix of human islets and affects the stiffness of the ECM, and determines cell fate [21, 24, 49]. Previous studies demonstrate that collagen IV significantly promotes cell survival in intact human islets [3, 6, 15] but may also decrease insulin production in beta cells [50] when present at high concentrations [6].

Combinations of collagens and polymeric biomaterials or other ECM molecules have been incorporated into transplanted grafts to create matrices with desired mechanical properties [16]. For example, collagen matrices have been incorporated into synthetic polymeric scaffolds to improve mechanical performance of the scaffolds [39, 50, 51]. Such an approach may also be used to enhance islet function, since collagen IV combined with specific laminin sequences has been found to improve glucose-stimulated insulin secretion (GSIS) in pancreatic islets [6, 52].

### Fibronectin

Fibronectin, a multifunctional component of ECM, facilitates cell attachment and cellular spreading by direct interaction with cells [53, 54]. The amino acid sequences of fibronectin interact with several cellular ligands. The best-known example is the tripeptide arginine-glycine-aspartic acid (RGD) receptor of fibronectin, which interacts with the β-I/A domain and is the synergy site in the adjacent fibronectin type III (FN3) repeat that interacts with the propeller domain [55, 56]. Accordingly, RGD has been incorporated into or applied on surfaces of numerous biomaterials [31]. Moreover, specific sections of the fibronectin RGD receptor can interact with α_4_β_1_, α_5_β_1_ and α_9_β_1_ integrins and with Ig superfamily cell-surface counter receptors, such as vascular cell adhesion molecule 1 (VCAM-1) [55]. One suggested strategy for improving functional cell survival in tissue engineering is to create several layers of oriented fibronectin to enhance the availability of its binding sites for cells [54, 57]. However, fibronectin can also interact with cells via non-integrin receptors, such as dystroglycan and syndecan [58, 59].

Fibronectin regulates several processes in islets via interaction with islet integrin and non-integrin receptors. It improves islet and beta cell function and, through transcriptional upregulation of the anti-apoptotic protein B cell lymphoma 2 (Bcl-2), has been demonstrated to enhance islet survival [6, 16, 60]. Lin et al showed that fibronectin stimulates beta cell proliferation and GSIS [61], whilst other have shown that fibronectin induces gene expression of differentiation markers for endocrine tissue, such as insulin 2, glucagon, *Pdx1* and *Pax6* [62]. Further, in vitro studies on porcine islets have demonstrated that a fibronectin-mimetic peptide can specifically bind to α_5_β_1_ integrin and increase matrix production and cell viability in isolated islets [60].

### Laminins

Laminins are heterotrimeric glycoproteins composed of α, β and γ polypeptide chains joined by disulfide bonds [63]. The specific expression and distribution of laminin isoforms in islets are not well understood [64]. However, recent studies report that laminins co-localise with α_6_ integrins in the developing pancreas and promote islet function in vitro [64]. Laminin-111 (composed of α_1_, β_1_ and γ_1_ chains) is the primary isoform present in the developing mouse pancreas [36, 65]. However, when mice reach adulthood, this is replaced by laminin-511, a trimer of the α_5_, β_1_, and γ_1_ isoform [64]. In human islets, laminin-411 (composed of α_4_, β_1_ and γ_1_ chains) and -laminin-511 have been found to be essential for beta cell proliferation and insulin transcription [66].

In terms of distribution, laminin-332 has been found to be present near the glucagon producing alpha cell [67], whilst laminin-511/521 is present in the double basement membrane layer of human islets [40]. Interactions with the islet cell membrane may not necessarily occur through integrins, as (like fibronectin) laminins may also bind to receptors of a non-integrin nature. For example, they may bind to dystroglycan to regulate assembly of the basal lamina [20] or induce beta cell differentiation and survival in fetal mouse pancreas [65, 68]. Most of the integrin-binding regions can bind to specific adhesive fragments of laminin [69], such as IKVAV, VAYI and IKLLI and laminin-111, which are all α_1_ chains [3, 70–73]. Other adhesive amino acid sequences of laminin, including YIGSR, PDSGR, RYVVLPR and LGTIPG, are present in the β_1_ chain [16, 20, 70, 71]. Although little is known about the interactions of these ligands with pancreatic islet cells, laminin adhesive sequences are reported to improve the function of pancreatic islets in vitro [3, 6]. Furthermore, laminins induce expression of islet-specific transcription factors and hormones, such as pancreatic and duodenal homeobox 1 (PDX1), insulin 1, insulin 2, glucagon, somatostatin and GLUT-2 [62]. They also activate protein kinase B (Akt) and extracellular signal-regulated kinase, (ERK), which are important regulators of cell metabolism and can induce differentiation of precursor cells into beta cells [61].

### Glycosaminoglycans

Glycosaminoglycans (GAGs) are linear sugar chains consisting of repeating units of disaccharides, hexosamine (glucosamine or galactosamine) and uronic acid [44]. Except for hyaluronic acid, these disaccharide chains are covalently linked to core proteins to form proteoglycans. Hyaluronic acid is localised in the ECM of pancreatic islets, whilst heparan sulfate proteoglycans (HSPGs; another class of GAGs) are concentrated in the intracellular space of beta cells [74, 75] and in the peri-islet basement membrane of islets in mice [19, 76]. In humans, the HSPG perlecan has been found to be present in beta cells from those with and without type 2 diabetes [77].

GAGs, particularly HSPGs, may also be involved in islet amyloid formation and cellular dysfunction [78]. For example, perlecan and agrin are HSPGs that exist in different isoforms and conformations in the pancreas. They are the primary carriers of heparan sulfate side chains in islets. Although the presence of perlecan and agrin in the islet basement membrane has not yet been elucidated [53], they are thought to dictate the composition of the vascular basement membrane, and also beta cell function [5, 75]. Specifically, in humans, perlecan is suggested to be involved in beta cell dysfunction. To support the role of these HSPGs in islet health, there is evidence that decreasing GAG synthesis might reduce islet amyloid formation [77–79]. Furthermore, decreasing HSPG levels or the addition of heparinase has been found to reduce amyloid formation [78]. In addition, a study by Ziolkowski et al suggests that the abundance of heparan sulfate was altered in islets and/or lymphoid tissue upon type 1 diabetes development [76].

### Fibrin

Fibrin, a complex matrix formed by polymerisation of fibrinogen, plays an important role in homeostasis and tissue repair [44]. Although fibrin is not a regular component of the ECM, it may be present as a temporary matrix that is replaced by other ECM molecules. Fibrin has many clinical applications [80, 81], including use as a biodegradable scaffold or glue to support islets after transplantation [82, 83]. It has also been applied as a delivery matrix for biomedical purposes, especially in combination with other biodegradable substances. The fact that fibrin has a matrix structure that is similar to the native pancreas makes it a candidate ECM protein for the support of long-term islet survival [81, 84, 85]. In recent studies, we have applied fibrin in polymeric scaffolds that serve as an artificial transplantation site for pancreatic islets under the skin [86]. This resulted in enhanced vascularisation and engraftment of islets, reducing the number of islets needed to achieve normoglycaemia in mice [86, 87]. This might be explained by specific interactions of fibrin with some integrins in islets, including α_v_β_1_, which is known to be important for the function of transplanted islets [88]. In vitro studies have demonstrated enhanced survival of insulin-producing cells when in contact with fibrin [61, 80, 81]. For example, Riopel et al cultured islets in fibrin and demonstrated improved beta cell function and survival, which was associated with regulation of focal adhesion kinase (FAK), ERK1/2 and Akt [80]. Fibrin can also upregulate α_v_β_3_ integrin expression, which prevents beta cell apoptosis [80, 83]. Thus, fibrin is an excellent candidate for exogenous addition to islet grafts to enhance their survival [86, 87].

## Cellular functions guided by ECM

As mentioned, combinations of ECM molecules play an important role in cell proliferation, differentiation and migration [32, 33] and, although functional aspects of ECM molecules have not been studied in detail in the endocrine pancreas, it is likely that cellular functions and the integrity of the pancreas is dependent on the ECM [20]. In one in vitro study, the addition of exogenous ECM enhanced rat beta cell proliferation in the presence of human growth hormone and the glucagon-like peptide-1 analogue liraglutide [89]. Another study found that islet heparan sulfate was involved in the regulation of postnatal islet growth and insulin secretion [76]. Specifically, in pancreatic islets, laminins α_4_ and α_5_ were found to be essential for normal beta cell adhesion, proliferation and insulin secretion [90]. On the other hand, however, in vitro studies on beta cell proliferation by Rutti et al indicated that specific ECM substratum or a similar natural basement membrane structure was necessary for human beta cell proliferation [91].

As mentioned above, ECM molecules convert their signal into a biological response via ligation to cellular receptors, such as integrins [92] and non-integrin receptors. Specific sequences in ECM molecules are responsible for this ligation. The RGD sequence, for example, binds to α_IIb_β_3_ and α_v_β_3_ integrin in the β subunit N-terminus [56, 93, 94]. RGD sites can be found in ECM proteins present in the endocrine pancreas, such as entactin, fibronectin, fibrinogen, laminin, vitronectin, von Willebrand factor and, in some cases, collagens [54]. Ligation of RGD to these ECM molecules can directly activate intracellular signalling pathways associated with prevention of apoptosis [16, 95]. Indeed, positive effects of RGD supplementation on islet cell survival have been observed by us and others [6, 16, 95].

The potency of manipulating ECM to achieve beneficial effects on pancreatic islet function is demonstrated by the stimulation of interactions between integrins and the ECM that modulate the expression of fibroblast growth factor receptor-1 (FGFR1) in beta cells, a receptor that regulates pathways involved in beta cell survival and function [20]. However, in addition to direct activation of signalling pathways via receptor interaction, ECM molecules (such as laminins), along with growth factors, cytokines, matrix metalloproteinases (MMPs) and other signalling molecules, can also regulate cellular functions through indirect pathways [96]. Two types of indirect mechanisms have been identified. One mechanism involves modulation of cytokine activity [97]; for example, in beta cells cultured on pancreas-specific ECM, a transient activation of NF-κB downstream activity and integrin engagement, as well as FAK activation are observed [98]. This leads to proliferation, enhanced cell survival and GSIS [3] and reduced cytokine-associated effects [12]. The other mechanism involves the ECM serving as a depot for growth factors that support tissue repair and homeostasis. This has not been studied extensively in islets but in the liver, for example, hepatocyte growth factor (HGF) is stored in the ECM in an inactive form. When the liver is damaged, the ECM is degraded by MMPs to release HGF, which stimulates hepatocyte proliferation [99, 100]. Whether these indirect mechanisms might also be involved in the pancreas remains to be demonstrated.

## ECM, MMPs and cellular receptors

**Fig. 1:**
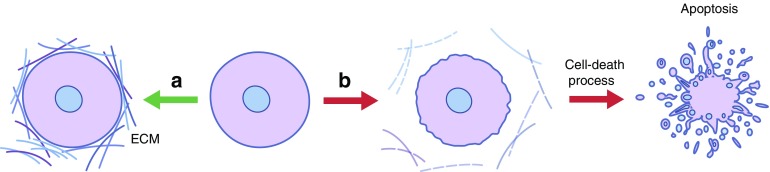
Cell function and survival is determined by ECM–cell interactions. (**a**) All cells require interaction with ECM for homeostasis of cellular functions. (**b**) Loss of ECM–cell interactions owing to mechanical forces or enzymatic digestion trigger cell-death processes, such as apoptosis. This figure is available as part of a downloadable slideset

Some believe that changes in ECM composition and ECM interaction with cells act as a morphogenetic language that is precisely interpreted by cells [32]. The sensing of embedded information in the ECM by specialised receptors at the cell surface has an important influence on cell behaviour. These receptors not only affect spatial differences in shape and function of cells but they also guide migration and intracellular processes, which is an often-ignored aspect of mechanical homeostasis in tissues [29]. The attachment of cells to ECM molecules can influence cell survival. Excessive mechanical force or other stressors, such as disturbances induced by enzymes, may influence ECM ligand–cell interactions and may trigger cell-death processes such as apoptosis [101] (Fig. 1). Manipulating ECM composition is therefore considered to be an effective approach to steer cellular function [3, 102, 103]. An example of this is the manipulation of hyaluronan (an inflammatory mediator of islet destruction) in the peri-islet and intra-islet environment [104].

In addition to adding ECM molecules, modulation or improvement of cellular function can be achieved by adding ECM-modulating MMPs, which carry out multiple functions, such as lysis of susceptible intracellular proteins in subcellular compartments [105]. A multitude of cytokines, such as TNF-α, IL-lβ, IL-17 and IL-18 [106–108], and chemokine ligand (CCL)2 (also known as monocyte chemoattractant protein -1 [MCP-1]), CCL3 (macrophage inflammatory protein [MIP]-1 α), and CCL5 (regulated on activation, normal T cell expressed and secreted [RANTES]) stimulate the release of monocyte MMP [109]. MMPs create the cellular environment required during development and morphogenesis by modulating the ECM [105]. In fact, an original criterion for an enzyme to be classified as an MMP was ‘the ability of the enzyme to proteolytically process at least one ECM protein’ [99]. However, it is now recognised that MMPs can cleave many other molecules besides ECM [110], including cytokines, chemokines, receptors, growth factors and cell adhesion molecules [111], thus influencing many biological processes. For example, IL-1β is cleaved into its active form by several MMPs, including MMP-2 and MMP-9, which are considered to be required for islet formation [100, 112]. Furthermore, growth factors such as fibroblast growth factors (FGFs), IGFs [113] and TGF-β [99] are known to be modulated by extracellular MMPs.

**Fig. 2:**
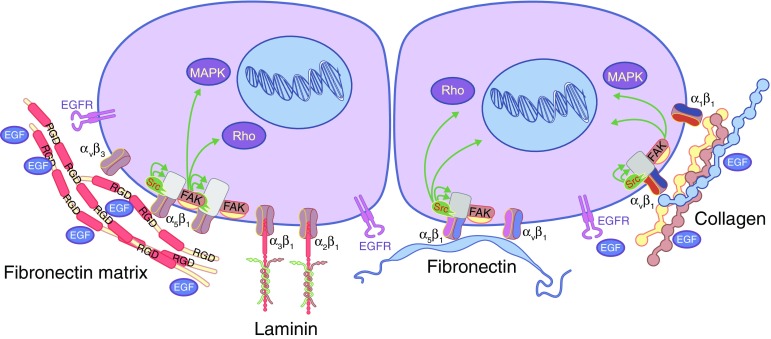
Summary of the current knowledge on ECM–cell interactions within islets. Integrin receptors bind to and interact with several ECM molecules in the pancreas, including collagen, RGD, fibronectin and laminin. Integrins, Src family kinases, and Rho GTPases are essential in mediating cellular responses downstream of ECM engagement. Stimulation of integrins initiate signalling cascades that ultimately affect the expression of genes influencing survival, growth and differentiation of cells. For example, FAK activation and subsequent activation of Akt and MAPKs can result from ECM–integrin interactions in the pancreas, whilst other integrin subunits crosstalk with growth factor receptors like EGFR. This figure is available as part of a downloadable slideset

Since integrins connect ECM molecules with the cytoskeleton via adapters, such as talin [114], and initiate signalling cascades that ultimately affect the expression of genes influencing survival, growth and differentiation of cells [114, 115], the effect of integrin stimulation on beta cell proliferation has also been explored. Integrin receptors bind to and interact with several ligands in the pancreas, such as collagen, RGD, fibronectin and laminin [20, 116]. The presence of α_3_, α_5_, α_v_, α_6_, β_1_, β_3_ and β_5_ integrin components have been reported in adult pancreatic islets [21, 117], α_3_, α_5_, α_6_ and β_1_ integrin in fetal pancreatic tissue [118, 119] and α_3_, α_v_, α_6_, β_1_ and β_5_ integrin subunits in endocrine pancreas cells [40, 117, 118]. Figure 2 summarises the current knowledge on interactions of integrins with ECM within islets. Stimulation of integrins leads to activation of FAK and subsequent activation of Akt and mitogen-activated protein kinases (MAPKs) [120]. Some integrin subunits, including α_v_, β_1_, β_4_, and β_7_, can crosstalk with growth factor receptors like EGF receptor (EGFR) [121]. The β_1_ subunit is of special interest for cell transplantation and tissue engineering since it has been shown to be important in maintaining stem cell function in several organs, including islets [115, 122, 123].

Islet integrins can be stimulated via different ECM molecules (Table 1). For example, it has been reported that α_6_β_1_ is involved in the transduction of a matrix signal that modulates beta cell function and enhances insulin secretion [124] and that interaction of this integrin with laminin-5 stimulates rat beta cell proliferation [124]. In other examples, ligands for the islet-specific α_3_β_1_ integrin include fibronectin, laminin, collagen I and collagen IV, yet only collagens I and IV promote rat INS-1 cell viability and proliferation [125]. Additionally, α_v_β_3_ binds tenascin C, which is a glycoprotein linked to promotion of tumour progression [126] whilst, dystroglycan has been implicated to play a role in laminin-111-induced beta cell differentiation and survival in the fetal mouse pancreas [20, 68]. Furthermore, Kaido et al [50] found that purified human beta cells contain α_v_β_1_, α_v_β_5_, and α_1_β_1_, suggesting that many of these integrins are localised in or on beta cells and that α_1_, α_v_ and β_1_ integrins might be important for creating and maintaining islet architecture. These interactions between ECM and integrins can induce both intracellular and extracellular processes [127]. Intracellular signalling activated by integrin–ECM interaction can alter the effects of cytokines on cells [128] and, hence, might be a novel tool for modulating the sensitivity of transplanted cells to avoid cytokine-associated cytotoxicity.

**Table 1:** Integrin receptors for ECM components in pancreatic islets

ECM component	Integrin subunits	Reference
Collagen	α_1_β_1_, α_2_β_1_, α_3_β_1_, α_v_β_1_, α_10_β_1_, α_11_β_1_	[18, 43, 45, 50, 56, 118, 119]
Fibronectin/fibrin	α_3_β_1_, α_4_β_1_, α_5_β_1_, α_4_β_7_, α_8_β_1_, α_v_β_1_, α_v_β_3_, α_v_β_5_, α_v_β_6_, α_IIb_β_3_	[18, 80, 93, 94, 118]
Laminin	α_1_β_1_, α_2_β_1_, α_3_β_1_, α_6_β_1_, α_7_β_1_, α_9_β_1_, α_v_β_3_, α_v_β_5_, α_v_β_8_, α_6_β_4_	[64, 67, 71, 73, 116–118, 144]
GAGs	α_2_β_1_, α_M_β_2_, α_IIb_β_3_, α_v_β_3_, α_4_β_1_, α_5_β_1_	[18, 19, 58]

## Immunomodulation and ECM

In addition to its direct interaction with cells, the ECM can serve as a reservoir for immune-active signalling molecules, such as cytokines and growth factors [29, 105], thereby acting in an immunomodulating capacity [129]. For example, when bound to fibronectin or laminin, cytokines (e.g. TNF-α) were found to improve the adhesion of T lymphocytes to fibronectin/laminin via integrin β_1_-dependent interactions [130].

Proteoglycans are the major ECM component involved in immune signalling. Further, perlecan and agrin [53, 131], located in the basement membranes surrounding the exocrine acini of adult mouse pancreas [19], are involved in the controlled release of cytokines and growth factors. Proteoglycans can serve as a binding site for IFN-γ and TGF-β [12, 132] and they are also implicated in interactions with heparin-binding cytokines, such as TNF-α. They can also act as a binding site for TGF-β; for example, a group of small proteoglycans, including decorin, biglycan and fibromodulin, regulate immune responses by binding TGF-β [133]. In an in vivo study, decorin inhibited TGF-β by immobilising it and preventing it from interacting with its cell surface signalling receptors [12].

Other matrix molecules, such as laminin-332, can mediate the synthesis of TGF-β1 and TNF-α [134]. Moreover, certain cells may enable activation of specific cytokines, such as IL-3, IL-7 and IFN-γ, by changing the composition of their cell-surface heparan sulfates [135, 136]. This process is fine-tuned by an interplay between the affinity of the receptor and the ligand cytokine/growth factor [137]. The dynamics of this process have been best studied using FGF-2, which binds to heparan sulfate chains at lower affinity than to its receptor [133].

The efficacy of cytokines can also depend on ECM molecules as co-receptors, or on ECM receptors molecules, such as integrins [137]. For example, simultaneous binding of growth factors/cytokines to their respective signalling receptor and to heparan sulfate chains forms the basis of dual receptor cytokine signalling [137]. Cytokine receptors are also required for the clustering of integrins, enabling effective signal transduction pathways [137, 138]. This clustering of receptors also applies to key cytokines involved in beta cell destruction in type 1 diabetes, such as IL-1β [138, 139]; interestingly the expression of ECM-associated genes (even at low levels) is associated with diminished IL-1β effects [10].

## Considerations for engineering and future applications of ECM in pancreatic islet transplantation

Human islets are currently isolated by application of enzyme mixtures, which contain collagenase, neutral protease, trypsin and clostripain [140]. As mentioned above, these mixtures selectively break down the connections between the exocrine and endocrine tissue, but also break down the islet vasculature and ECM [2, 141]. For example, following administration via the ductal circuit collagenase may enter the islets and destroy intra-islet ECM [2]. Several studies have also shown detrimental effects of enzymatic islet isolation on peripheral islet ECM, such as on collagens [2, 6] and laminins [119]. Collagenases digest several collagen types, such as types I, III, IV and V [25]. This has a dramatic impact on cell viability [27]. The enzymes also damage intracellular stores of the GAG heparan sulfates [22], with a predictable impact on islet transplant outcomes.

**Fig. 3:**
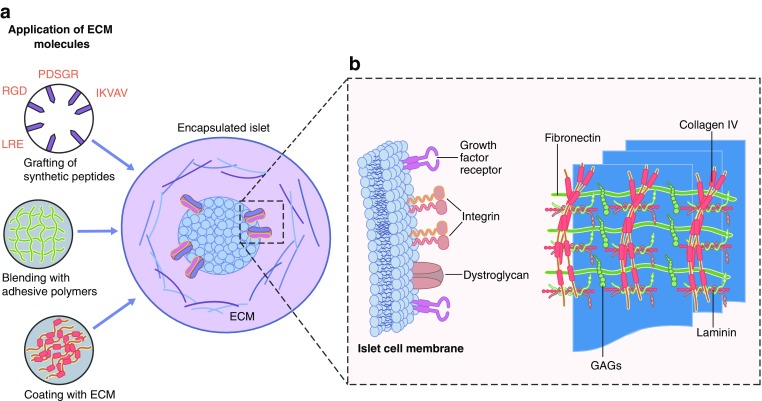
Supplementation of ECM in capsules for immuno-isolation of pancreatic islets enhance functional survival of the graft. (**a**) Specific ECM molecules may be added to islets before transplantation to enhance the functional survival of islets. For example, collagen IV and laminin sequences, such as RGD, LRE and PDSGR, have positive effects on the function of isolated human islets. (**b**) Supplemented ECM can mimic/interact with various molecules on the islet cell membrane. Immuno-isolation of islets by encapsulation has been used to demonstrate that ECM supplementation can promote islet cell survival. Encapsulation of islets in an immunoprotective but semipermeable membrane allows for successful transplantation of islets without the need for immunosuppression. This figure is available as part of a downloadable slideset

A possible strategy for enhancing the functional survival of islets is to add specific ECM molecules before transplantation. Recent studies have demonstrated that collagen IV and laminin sequences, such as RGD, LRE and PDSGR, have positive effects on the function of isolated human islets [6, 15, 16, 23]. An emerging field, in which we recently proved the principle of applying ECM supplementation for islet cell survival, is the immuno-isolation of islets by encapsulation (Fig. 3). Encapsulation of islets in an immunoprotective but semipermeable membrane allows for successful transplantation of islets without the need for immunosuppression [142]. One obstacle to this application is the loss of islets in the immediate transplant period, which can amount to 60% of the graft in the first 2 weeks after implantation [142, 143]. Recently, we demonstrated that the enzyme preparation has a huge influence on both islet cell survival and the immunogenicity of the tissue [2]. Islets isolated from the pancreas using a less-favourable enzyme mixture produced less insulin upon glucose challenge but more IFN-γ-inducible protein 10 (IP-10), growth related oncogene-α (GRO-α), MIP-1 and -2, RANTES, and cytokines, including MCP-1. This resulted in a twofold lower graft survival time than islets extracted using a different collagenase mixture [2].

In an attempt to enhance the survival of encapsulated pancreatic islets in alginate-based microcapsules, we investigated the impact of single ECM molecules and combinations of ECM molecules. We used bioactive sequences of ECM molecules instead of full proteins as they can be produced synthetically and are associated with fewer variations than ECM components from biological origin. An interesting observation was that not all ECM components had beneficial effects and effects vary depending on the concentration of ECM components. For example, excessively high concentrations of collagen IV had detrimental effects on GSIS [3, 6]; whilst, concentrations of collagen IV that mimicked physiological conditions in islets in vivo supported insulin secretion [29]. When combined with collagen IV, out of several ECM molecules, only RGD, LRE and PDSGR were found to have a positive impact on the function of pancreatic islets [6]. These studies highlight that there is still much to learn about the role of ECM in the biology and functional survival of pancreatic islets and that a stepwise approach is necessary to select beneficial ECM components. At the same time, it also demonstrates that supplementation with ECM components, as used in other fields, including tissue engineering, is a feasible approach to enhance pancreatic islet cell survival and graft function.

## Conclusion

Overall, it is clear that the manipulation of ECM may promote successful islet transplantation. However, there are still many questions left to be answered with regard to the type of ECM molecules that are beneficial for islet graft survival. To facilitate a response to these questions, however, we must first elucidate the mechanisms by which these ECM molecules enhance the survival and function of pancreatic islets. Importantly, these finding may aid with the development of future treatments for type 1 diabetes that will help to improve the life expectancy and quality of patients with type 1 diabetes.

## Electronic supplementary material

ESM (PPTX 901 kb)

## Abbreviations

CCL: Chemokine ligand
ECM: Extracellular matrix
EGFR: EGF receptor
ERK: Extracellular signal-regulated kinase
FAK: Focal adhesion kinase
FGF: Fibroblast growth factor
FGFR1: Fibroblast growth factor receptor-1
GAG: Glycosaminoglycan
GSIS: Glucose-stimulated insulin secretion
HGF: Hepatocyte growth factor
HSPG: Heparan sulfate proteoglycans
MAPK: Mitogen-activated protein kinase
MCP-1: Monocyte chemoattractant protein -1
MIP: Macrophage inflammatory protein
MMP: Matrix metalloproteinase
RANTES: Regulated on activation, normal T cell expressed and secreted
RGD: Arginine-glycine-aspartic acid
