# Supplementary material for: Extracellular matrix molecules and their potential contribution to the function of transplanted pancreatic islets
Source: Diabetologia. 2018 Jan 6;61(6):1261–72. doi: 10.1007/s00125-017-4524-8 (PMC6449002; doi:10.1007/s00125-017-4524-8)
Supplement: Supplementary file 1 — (PPTX 901 kb) [file 125_2017_4524_MOESM1_ESM.pptx]

## Slide 1
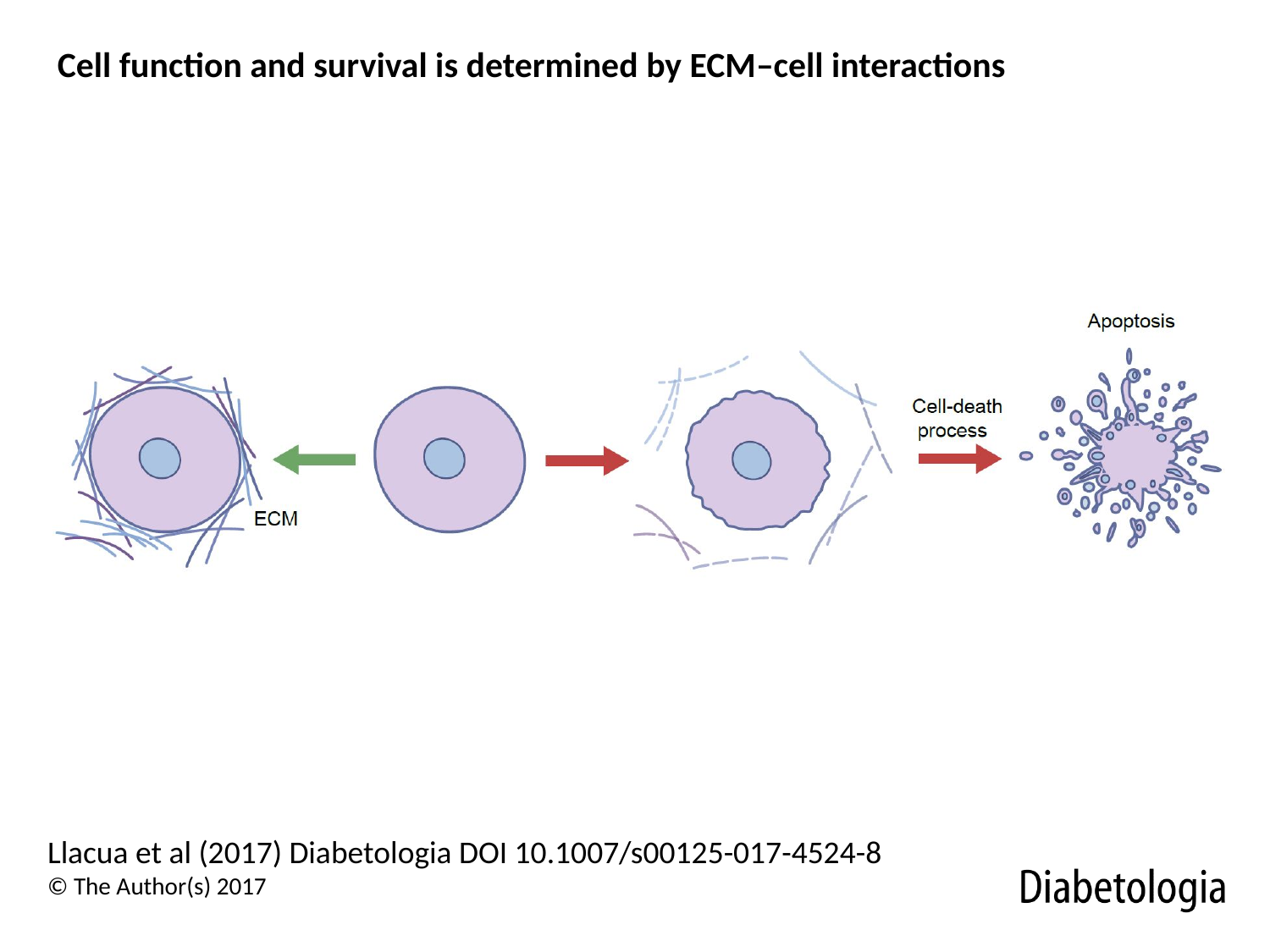

Cell function and survival is determined by ECM–cell interactions
Llacua et al (2017) Diabetologia DOI 10.1007/s00125-017-4524-8
© The Author(s) 2017

## Slide 2
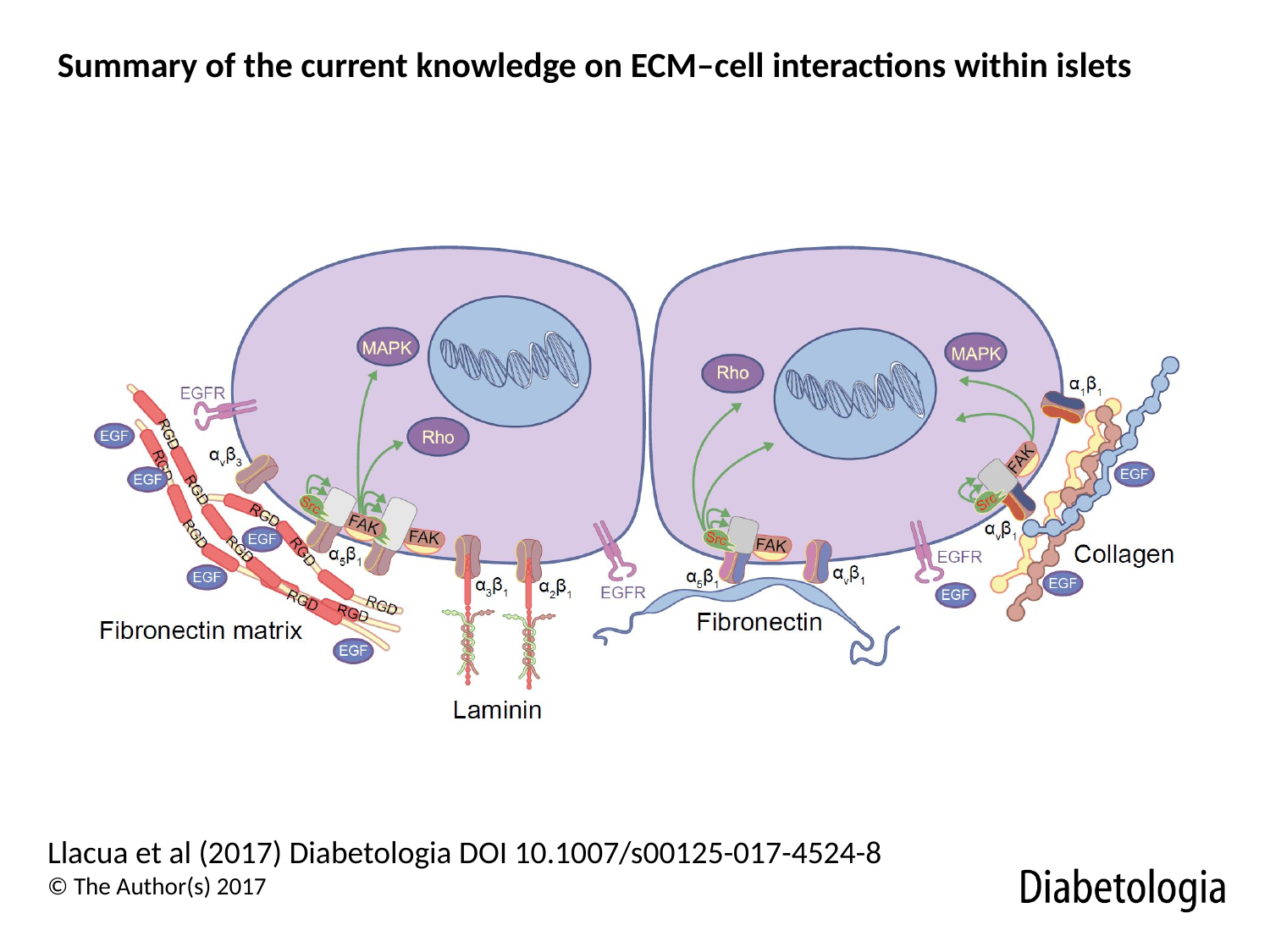

Summary of the current knowledge on ECM–cell interactions within islets
Llacua et al (2017) Diabetologia DOI 10.1007/s00125-017-4524-8
© The Author(s) 2017

## Slide 3
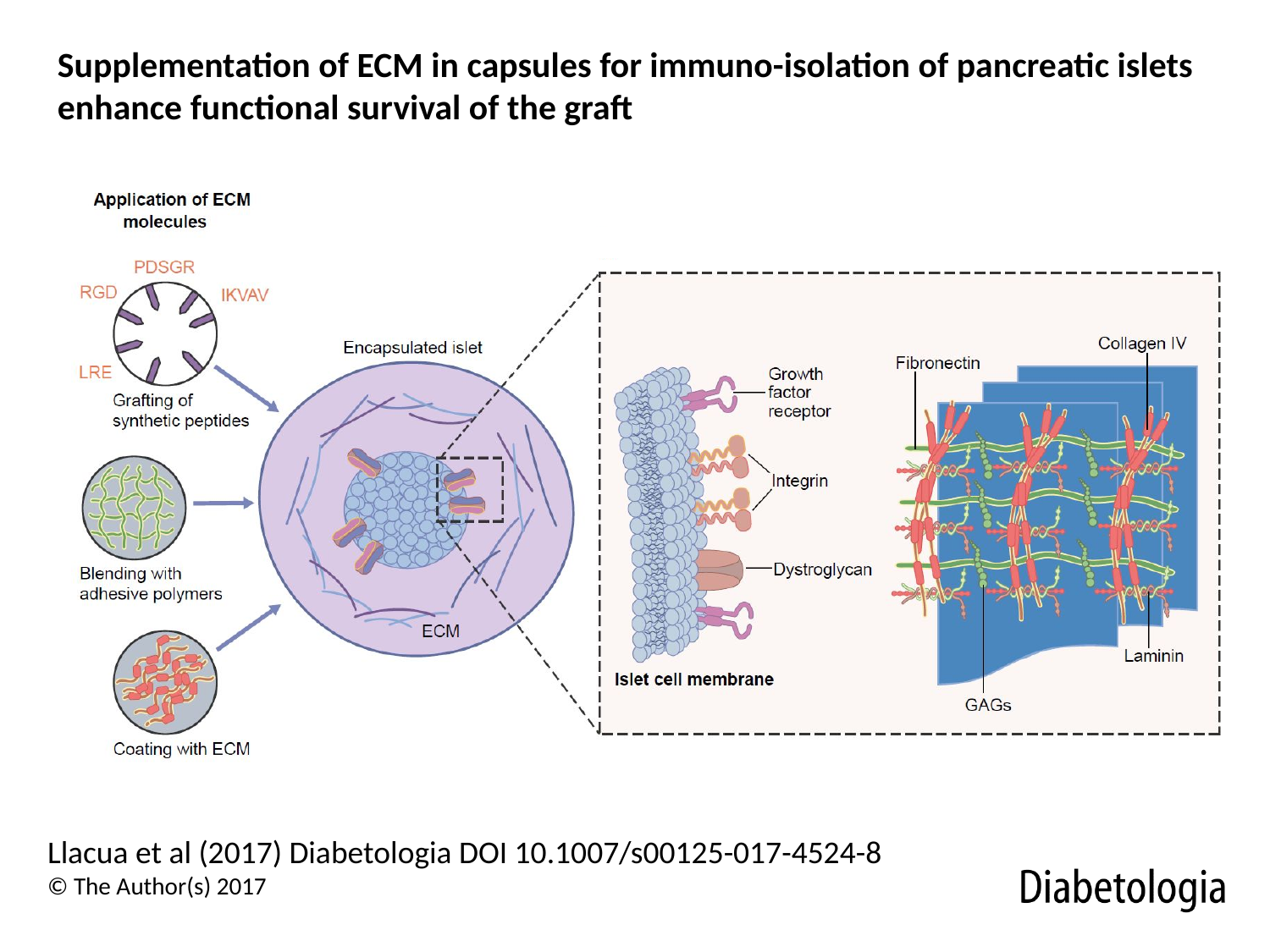

Supplementation of ECM in capsules for immuno-isolation of pancreatic islets enhance functional survival of the graft
Llacua et al (2017) Diabetologia DOI 10.1007/s00125-017-4524-8
© The Author(s) 2017
